# Structural insights into human ABCD3-mediated peroxisomal acyl-CoA translocation

**DOI:** 10.1038/s41421-024-00722-8

**Published:** 2024-09-03

**Authors:** Yang Li, Zhi-Peng Chen, Da Xu, Liang Wang, Meng-Ting Cheng, Cong-Zhao Zhou, Yuxing Chen, Wen-Tao Hou

**Affiliations:** 1 Department of Endocrinology, Institute of Endocrine and Metabolic Diseases, The First Affiliated Hospital of USTC, and Center for Advanced Interdisciplinary Science and Biomedicine of IHM, Division of Life Sciences and Medicine, University of Science and Technology of China, Hefei, Anhui China; 2 Biomedical Sciences and Health Laboratory of Anhui Province, University of Science and Technology of China, Hefei, Anhui China

**Keywords:** Cryoelectron microscopy, Protein folding

## Abstract

Human ABC transporters ABCD1–3 are all localized on the peroxisomal membrane and participate in the β-oxidation of fatty acyl-CoAs, but they differ from each other in substrate specificity. The transport of branched-chain fatty acids from cytosol to peroxisome is specifically driven by ABCD3, dysfunction of which causes severe liver diseases such as hepatosplenomegaly. Here we report two cryogenic electron microscopy (cryo-EM) structures of ABCD3 bound to phytanoyl-CoA and ATP at resolutions of 2.9 Å and 3.2 Å, respectively. A pair of phytanoyl-CoA molecules were observed in ABCD3, each binding to one transmembrane domain (TMD), which is distinct from our previously reported structure of ABCD1, where each fatty acyl-CoA molecule strongly crosslinks two TMDs. Upon ATP binding, ABCD3 exhibits a conformation that is open towards the peroxisomal matrix, leaving two extra densities corresponding to two CoA molecules deeply embedded in the translocation cavity. Structural analysis combined with substrate-stimulated ATPase activity assays indicated that the present structures might represent two states of ABCD3 in the transport cycle. These findings advance our understanding of fatty acid oxidation and the molecular pathology of related diseases.

## Introduction

Peroxisomes are essential organelles in all eukaryotic cells, including mammals, plants and unicellular eukaryotes. In human cells, peroxisomes are involved in diverse metabolic reactions, including oxidation of fatty acid^1^, synthesis of bile acid^2,3^ and docosahexaenoic acid (DHA)^4^, metabolism of reactive oxygen species and reactive nitrogen species (ROS/RNS)^5^. In particular, β-oxidation of fatty acids is a common property of all types of peroxisomes^5^.

**Fig. 1:**
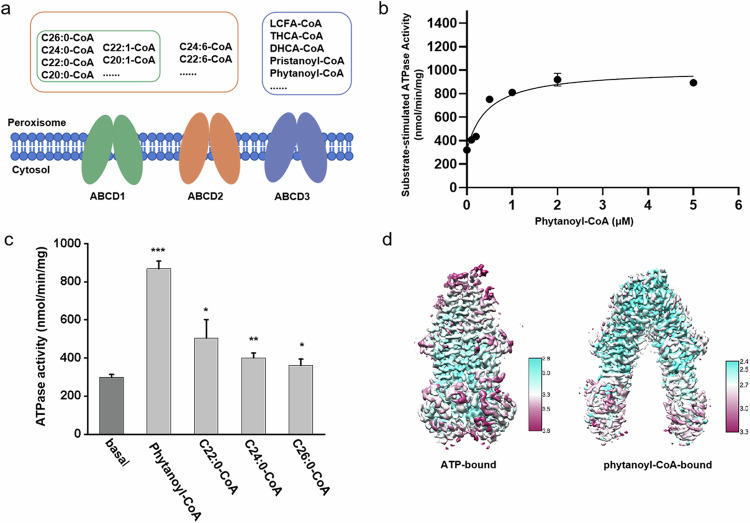
Substrate specificity and structure determination of hABCD3. **a** A diagram showing the substrate specificity of ABCD1–3. All three transporters locate on the peroxisome membrane and are indicated as a pair of ellipses in different colors. Each receptor’s corresponding substrates are highlighted with a rectangle of the same color as the receptor. DHCA-CoA/THCA-CoA, di-/tri-hydroxycholestanoyl acyl-CoA. **b** Substrate concentration-dependent ATPase activity of chABCD3 in the detergent of LMNG + CHS with 2 mM ATP upon addition of phytanoyl-CoA. The data points were fitted with a Michaelis-Menten equation. **c** ATPase activity of chABCD3 in the presence of various fatty acyl-CoAs. All data points for **b** and **c** represent means of three independent measurements in the detergent of LMNG + CHS. Error bars represent the means ± SD. Student’s *t*-test was used in the comparison of the basal ATPase activity and substrate-stimulated ATPase activity. The *P* values of phytanoyl-CoA, C22:0-CoA, C24:0-CoA, C26:0-CoA are 0.0000436, 0.0417, 0.00736 and 0.0672, respectively. *P < 0.05, **P < 0.01, ***P < 0.001. **d** Refined cryo-EM maps of two ABCD3 structures. The cryo-EM maps are colored by UCSF ChimeraX 1.2.5 according to the local resolution of the cryo-EM estimated by cryoSPARC 3.2.

Fatty acids are a class of carboxylic acids with long aliphatic chains. They are not only important dietary sources of fuel for humans but also pivotal structural components of cell membranes^6,7^. Fatty acids are obtained either through endogenous synthesis or from daily food. In humans, free fatty acids are usually bound to the albumin proteins in the blood and transported to various tissues throughout the human body^8^. In the cell, fatty acids are first activated by acyl-CoA synthetase to acyl-CoA^9^, and subsequently translocated into mitochondria or peroxisomes, where they are subjected to β-oxidation for providing the energy and/or substances of further cellular metabolism^10^. Particularly, the very long-chain fatty acyl-CoA (VLCFA-CoA) and branched-chain fatty acyl-CoA (BCFA-CoA) are selectively transported into the peroxisome by ATP-binding cassette (ABC) transporters, including adrenoleukodystrophy protein (ALDP/ABCD1)^11^, ALDP-related protein (ALDRP/ABCD2)^12^, and the 70-kDa peroxisomal membrane protein (PMP70/ABCD3)^13^. These three ABC transporters belong to the ABCD subfamily, which utilizes the energy of ATP binding/hydrolysis to transport the substrate against its concentration across membranes^14^. Despite all localizing on the peroxisomal membrane, these transporters differ from each other in substrate preference^2,15–17^. ABCD3 specifically transports CoA esters of dicarboxylic acid, BCFA, bile acid intermediates: di- or tri-hydroxycholestanoic (DHCA or THCA)^2^, whereas ABCD1 and ABCD2 partly overlap in the substrate preference (Fig. 1a), which are CoA esters of saturated, monounsaturated and polyunsaturated fatty acids^18^. Due to the distinct substrate specificity, dysfunction of these transporters would cause various diseases. Pathogenic variants of ABCD1 lead to X-linked adrenoleukodystrophy, a progressive genetic disorder affecting the adrenal glands, spinal cord, and central nervous system^19,20^. The mutation in the *ABCD3* gene was reported to lead to accumulation of the peroxisomal C27-bile acid intermediates DHCA and THCA in plasma, and consequently causing severe liver diseases such as hepatosplenomegaly^2^.

Recently, five groups independently reported ABCD1 structures in different states^21–25^, which altogether delineate snapshots of a complete transport cycle of ABCD1 and provide insight into its substrate specificity. However, a detailed translocation pathway of BCFA-CoAs remains ambiguous. Therefore, structural information on ABCD3 is needed for better understanding of its specific substrate recognition and transport mechanism, which will also increase our knowledge of related human diseases.

Here we show the molecular structures of human ABCD3 (termed hABCD3 for short hereafter) determined in two distinct functional states by single-particle cryogenic electron microscopy (cryo-EM): the substrate- and ATP-bound structures at 2.9 Å and 3.2 Å, respectively. Combined with site-directed mutagenesis followed by substrate-stimulated ATPase activity assays, our data not only unveil the two states of the BCFA-CoA transport cycle propelled by ABCD3, but also offer biochemical proof regarding the binding pattern of acyl acids. These results disclose an underlying translocation cycle involving multiple intermediate states, which might also apply to ABCD1/2 and homologs, and shed light on the pathogenesis of the related human diseases.

## Results

### Biochemical characterization and structure determination of chABCD3

We initially over-expressed the full-length hABCD3 in HEK293F cells, but failed to purify sufficient amount of recombinant protein for further study. Alternately, we obtained a much higher yield for PMP2 from *Caenorhabditis elegans*, a 61.5% sequence-identical homolog of hABCD3 (Supplementary Fig. S1a)^26^. Sequence alignment indicated that hABCD3 and PMP2 differ from each other mainly in the N-terminal segment (Supplementary Fig. S1a), which may be involved in the subcellular location^26–28^. Therefore, we rationally constructed a chimeric version of ABCD3 (termed chABCD3), with the N-terminal 50 residues of PMP2 fused to the core domains of hABCD3. As expected, the expression level of chABCD3 is ~10-fold higher than that of hABCD3 (Supplementary Fig. S1b).

The ATPase activity assays showed that the purified chABCD3 protein possessed a robust ATPase activity in the buffer containing lauryl maltose neopentyl glycol (LMNG) and cholesteryl hemisuccinate (CHS), with *K*_m_ and *V*_max_ values of 0.16 mM and 343.9 nmol Pi/min/mg protein, respectively (Supplementary Fig. S1c). Of note, the ATPase activity of chABCD3 is comparable to that of hABCD3, whose *K*_m_ and *V*_max_ values are 0.24 mM and 288.1 nmol Pi/min/mg protein, respectively (Supplementary Fig. S1d). In contrast, the classic loss-of-function variant generated by replacing the catalytic glutamine of chABCD3 (E596Q) exhibited an ATP hydrolysis activity with *K*_m_ and *V*_max_ values of 0.19 mM and 107.3 nmol Pi/min/mg protein, respectively (Supplementary Fig. S1c), showing a comparable *K*_m_, but a *V*_max_ value of ~1/3 to the wild type (WT). Similar ATPase activity of EQ mutants in some ABC transporters has been reported previously^23,29^. Remarkably, the substrate phytanoyl-CoA (a BCFA-CoA, Supplementary Fig. S1f), stimulated the ATPase activity of chABCD3 in a concentration-dependent manner with the EC_50_ (concentration for 50% of maximal effect) value at ~0.46 μM and *V*_max_ of 1007.0 nmol Pi/min/mg protein (Fig. 1b). In addition, the assays also revealed a Hill coefficient of 1.7 (Supplementary Fig. S1e), implying that one ABCD3 molecule can bind two molecules of phytanoyl-CoA in a cooperative way. In contrast, the addition of any VLCFA-CoA (either C22:0-CoA, C24:0-CoA or C26:0-CoA) yielded much less stimulation of the ATPase activity (Fig. 1c; Supplementary Fig. S1f), suggesting that phytanoyl-CoA is the most favored substrate of chABCD3 among these tested acyl-CoAs, which is consistent with a previous report^2^. This also indicated that our sample of chABCD3 is in a physically relevant state.

To optimize the samples for single-particle cryo-EM analysis, we tested various detergent combinations and found that chABCD3 behaved the best in glyco-diosgenin (GDN) and digitonin (Supplementary Fig. S1g). Correspondently, the ATPase activities were attenuated in both detergents, compared to those in the LMNG + CHS buffer (Supplementary Fig. S1g). Eventually, we solved two structures of chABCD3 (Fig. 1d): the phytanoyl-CoA-bound form at 2.9 Å (Supplementary Fig. S2 and Table S1) in digitonin, and the ATP-bound form at 3.2 Å in GDN (Supplementary Fig. S3 and Table S2). Notably, the fused N-terminal segment of PMP2 is invisible in the two structures, most likely due to its high flexibility. This indicated that the two structures indeed represent the core structure of hABCD3; thus, we used ABCD3 for short to describe the structural information.

### Structural features of ABCD3

**Fig. 2:**
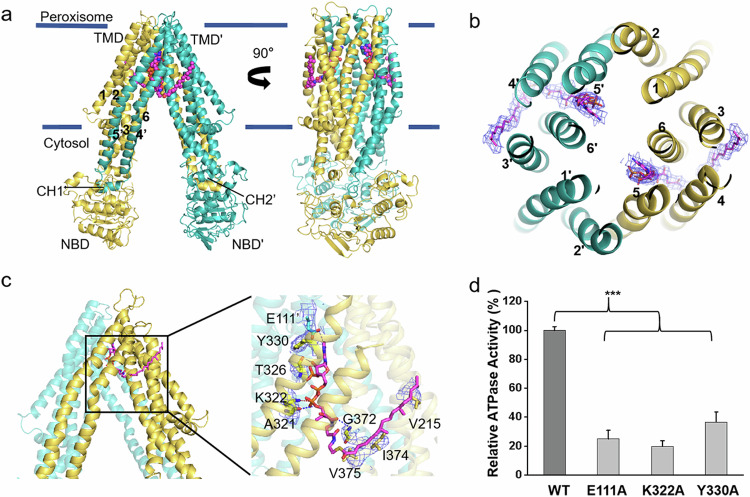
Structure of substrate-bound ABCD3 and the substrate-binding sites. **a**, **b** Side (**a**) and top (**b**) views of the overall structure of phytanoyl-CoA-bound ABCD3. Two subunits of ABCD3 are colored in yellow and teal, respectively. The peroxisome membrane boundaries are indicated by two blue lines. The two phytanoyl-CoA molecules are shown as magenta sticks. The density maps of phytanoyl-CoA molecules are shown as blue mesh, and are contoured at 5σ. **c** The phytanoyl-CoA-binding residues are shown as sticks, and hydrogen bonds (≤ 3.5 Å) and salt bridges (≤ 4.0 Å) are indicated as blue dotted lines. The hydrophobic residues surrounding the fatty acyl chain within 4.5 Å are shown as sticks. The density map of residues shown as blue mesh are contoured at 7σ. **d** Relative ATPase activities of chABCD3 and mutants in the detergent of LMNG + CHS with 2 mM ATP upon addition of phytanoyl-CoA. The relative activity represents the substrate-stimulated ATPase activity of chABCD3 or its variants each harboring a single mutation in the residues at the substrate-binding site. Each data point is the average of three independent experiments, and error bars represent the means ± SD. Student’s *t*-test was used for the comparison of the phytanoyl-CoA-stimulated ATPase activites between WT chABCD3 and its variants. The *P* values of E111A, K322A, Y330A are 0.0000802, 0.0000169, and 0.000278, respectively. *P < 0.05, **P < 0.01, ***P < 0.001.

The phytanoyl-CoA-bound structure of ABCD3 was solved with the addition of 0.5 mM phytanoyl-CoA during sample preparation. The overall structure exhibits a two-fold symmetric homodimer, with each subunit containing a nucleotide-binding domain (NBD) and a transmembrane domain (TMD) (Fig. 2a; Supplementary Fig. S4a). The phytanoyl-CoA-bound ABCD3 adopts an inward-facing conformation open to cytosol, similar to the apo and substrate-bound structures of ABCD1^21^. Each TMD of ABCD3 consists of six transmembrane helices (TMs), which are tightly packed against each other in the peroxisomal membrane leaflet, but separated into two TM bundles in the cytosolic membrane leaflet and further extend to the cytosol. The helices TM4 and TM5 from one TMD are swapped to the opposite TMD, i.e., domain swapping, which is a typical feature of type-IV ABC transporters^29^. Two pairs of coupling helices from the TMDs are embedded in the grooves on the NBDs, coupling the conformational changes between TMDs and NBDs.

The NBDs of ABCD3 possess a canonical NBD fold of ABC transporter, but without the crossover helix pair as found in ABCD1 (Fig. 2a). Sequence alignment revealed that the sequence corresponding to the C-terminal helix in both ABCD1 and ABCD2 is absent in ABCD3 (Supplementary Fig. S4b). It was proposed that this C-terminal helical crossover in ABCD1 may facilitate the dimerization of two separated NBDs, corresponding to a very low basal ATPase activity^21^. Indeed, the basal ATPase activity of ABCD3 without the C-terminal helical crossover is much higher than that of ABCD1.

### Two phytanoyl-CoA molecules symmetrically bind to ABCD3

During structural refinement, we observed two symmetric cryo-EM densities in the TMDs that could be unambiguously fitted with two phytanoyl-CoA molecules (Supplementary Fig. S5c). Unlike our previously reported substrate-bound ABCD1 structure in which each C22:0-CoA molecule crosslinks two TMDs^21^, each phytanoyl-CoA molecule solely binds to one TMD. In detail, the hydrophilic head group protrudes into the central translocation cavity, whereas the acyl tail is buried in the fenestration formed by TM3, TM4, TM5, and TM6 (Fig. 2b, c). Specifically, the adenine ring of the 3′-phospho-ADP moiety is stabilized by Glu111 from TM2′ (TM2 from the other subunit) via a hydrogen bond, in addition to the π–π interaction between Tyr330 from TM5 and the adenine ring. The 3′-phosphophate group of the ribose forms a hydrogen bond with the main chain of Thr326 from TM5, and the β-phosphophate of the diphosphate forms a salt bridge with Lys322 from TM5. In addition, the pantothenic acid moiety forms two hydrogen bonds with the main chains of Ala321 from TM5 and Gly372 from TM6, respectively. The branched chain fatty acid tail forms hydrophobic interactions with Val215 from TM3, in addition to Ile374 and Val375 from TM6 (Fig. 2c).

Site-directed mutagenesis combined with ATPase activity assays indicated that the chABCD3 variants with single mutation of the substrate-binding residues (E111A, K322A, Y330A), supplying either the polar or non-polar interaction with CoA, displayed significantly decreased substrate-stimulated ATPase activity, compared to the WT (Fig. 2d). In sum, the extensive polar and hydrophobic interactions ensure a highly specific binding pocket toward CoA and fatty acyl chain of phytanoyl-CoA, respectively. Moreover, multiple-sequence alignment revealed that most of these substrate-binding residues are conserved in ABCD3 and homologs (Supplementary Fig. S5d).

### Structural comparison revealed two intermediate states of substrate translocation

**Fig. 3:**
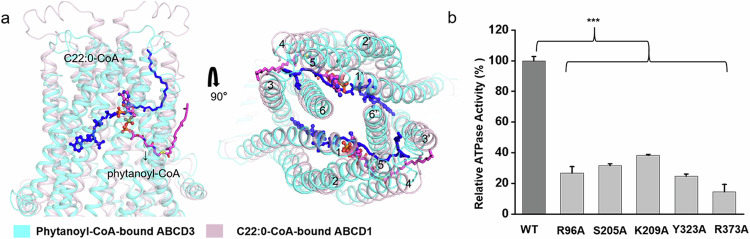
Comparison of phytanoyl-CoA-bound ABCD3 structure with the C22:0-CoA-bound ABCD1 structure (PDB code: 7VZB). **a** Side and top views of the superposition of the phytanoyl-CoA-bound ABCD3 (teal) against the C22:0-CoA-bound ABCD1 (lightpink). TMs are sequentially numbered and shown as cartoons. The phytanoyl-CoA molecules are shown as magenta sticks, and the C22:0-CoA molecules are shown as blue sticks. **b** Relative ATPase activities of chABCD3 and mutants in the detergent of LMNG + CHS with 2 mM ATP upon addition of phytanoyl-CoA. The relative activity represents the substrate-stimulated ATPase activity of chABCD3 or its mutants each harboring a single mutation of the conserved residues at the CoA substrate-binding site found in ABCD1. Each data point is the average of three independent experiments, and error bars represent the means ± SD. Student’s *t*-test was used for the comparison of the phytanoyl-CoA-stimulated ATPase activites between WT chABCD3 and its variants. The *P* values of R96A, S205A, K209A, Y323A, R373A are 0.0000298, 0.00000464, 0.00000578, 0.00000386 and 0.0000235, respectively. *P < 0.05, **P < 0.01, ***P < 0.001.

Similar to our recently reported structure of C22:0-CoA-bound ABCD1 (PDB code: 7VZB)^21^, the present phytanoyl-CoA-bound ABCD3 structure also exhibits an inward-facing conformation towards the cytosol side. Superposition of phytanoyl-CoA-bound ABCD3 structure against C22:0-CoA-bound ABCD1 revealed a root-mean-square-deviation of 6.348 Å over 1056 C_α_ atoms (Supplementary Fig. S6a), with a different substrate binding pattern. In ABCD1, the 3′-phospho-ADP portion of CoA binds to one TMD, while the pantothenic acid moiety extends from one TMD to the opposite TMD′ across the transport cavity (Supplementary Fig. S6b). Structural comparison indicated that the substrates are deeply buried in the TMDs of ABCD1, with the CoA moiety pointing towards NBDs (Fig. 3a). Specifically, one C22:0-CoA molecule interacts with TM1, TM2, TM3, TM6 of one TMD and TM3′, TM4′, TM5′, TM6′ of the other TMD, while one phytanoyl-CoA molecule only interacts with 4 TMs, indicating that we captured a state at which phytanoyl-CoA has not completely entered ABCD3. Considering the shared CoA moiety in C22:0-CoA and phytanoyl-CoA, and that most CoA-binding residues in ABCD1 are also highly conserved in ABCD3 according to sequence alignment (Supplementary Fig. S6b, c), we speculated that the two structures represent two intermediate states of one shared substrate translocation pathway. To further validate this hypothesis, we generated variants of ABCD3 with single mutation of the CoA-binding residues deduced from the structure of ABCD1. Despite remaining a basal ATPase activity comparative to the WT, the variants R96A, S205A, K209A, Y323A and R373A displayed abolished ATPase activity stimulated by phytanoyl-CoA (Fig. 3b).

### Upon ATP binding, ABCD3 adopts an open conformation towards the peroxisome

**Fig. 4:**
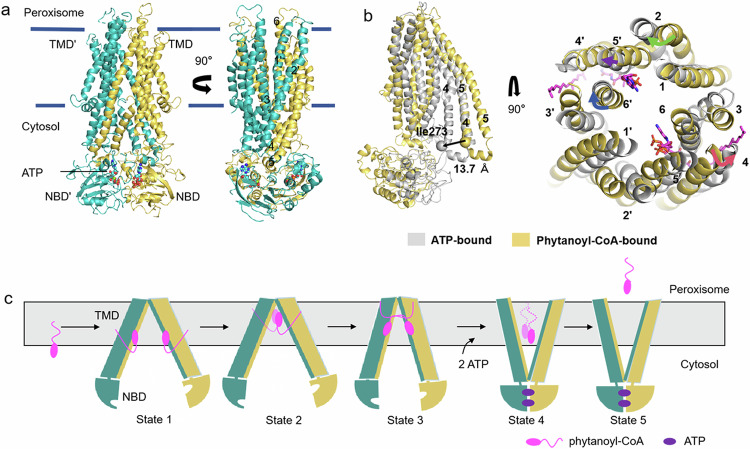
Structure of ATP-bound ABCD3 and structural superposition with the phytanoyl-CoA-bound ABCD3. **a** Cartoon representation of ATP-bound ABCD3. Two subunits of ABCD3 are colored in yellow and teal, respectively. ATP molecules are displayed as teal spheres. TMs are sequentially labeled. **b** Side and top views of the superposition of the ATP-bound ABCD3 (gray) against the phytanoyl-CoA-bound ABCD3 (yellow). TMs are sequentially numbered, and phytanoyl-CoA molecules are shown as magenta sticks. Cα atoms of Ile273 are displayed as spheres. The shifts of TM2, TM4, TM5′ and TM6′ are indicated by green, red, purple and blue arrows, respectively. **c** Proposed snapshots of the translocation cycle for the ABCD3-mediated phytanoyl-CoA translocation. At first, the CoAs enter the translocation cavity by diffusion while the fatty acid moieties stay in the membrane approaching ABCD3 (State 1). During the entry process, the CoA moiety points towards the peroxisomal space (State 2). After bound to the next binding sites, phytanoyl-CoA fully enters, with its CoA moiety pointing to the cytosol side, and the fatty acid is embraced by more TMs (State 3). The fully bound substrate triggers conformational change of ABCD3, facilitating the binding of ATP and a further moving of the CoA moiety towards the cytosol (State 4). After the hydrolysis of ATP, phytanoyl-CoA molecules are fully transported. Alternately, it is possible that phytanoyl-CoA is digested into CoA and phytanic acid, after which the phytanic acid is translocated into the peroxisome while CoA is released (State 5).

In order to capture the substrate release state of ABCD3, we prepared the cryo-EM sample with ABCD3 E596Q mutant via adding 10 mM ATP/Mg^2+^ and 0.5 mM phytanoyl-CoA, and solved an ATP-bound ABCD3 structure at a resolution of 3.2 Å with C2 symmetry (Supplementary Fig. S7a–c). In this structure, the two NBDs dimerize in a “head-to-tail” mode. The two TMDs pack against each other at the cytosol side, while separating at the peroxisomal matrix side, forming a narrow transport cavity to peroxisome (Fig. 4a). At the interface of two NBDs, two ATP molecules are sandwiched by the Walker A motif from one NBD and the ABC signature motif from the other (Supplementary Fig. S8a). In consequence, the conformational changes in NBDs are transferred to TMDs via two pairs of coupling helices, and eventually make the ATP-bound ABCD3 exhibit an outward-facing conformation (Fig. 4a). When comparing the ATP- and phytanoyl-CoA-bound ABCD3 structures, the ATP-bound structure displays a scissor-like movement, resulting in an enlarged exit of the translocation cavity (Fig. 4b). Specifically, TM4 and TM5 are split into two short helices due to the conformational changes, and the first half (lower half in Fig. 4b) moves towards the translocation cavity by ~13.7 Å as measured by the I273 C_α_ atoms. In contrast, TM2, TM6, and the second half (upper half in Fig. 4b) of TM4 and TM5 tilt away from the translocation cavity, leading to an expanded exit of the translocation cavity resulting from the binding of ATP, which is a state ready for substrate release (Fig. 4b).

## Discussion

Although phytanoyl-CoA was added during sample preparation for the structure analysis of ATP-bound ABCD3, we did not observe clear density for the full-length substrate, but a pair of extra densities in the translocation cavity (Supplementary Fig. S8b). After the trial of solving this structure under C1 symmetry, the resolutions of the densities were improved, although the overall resolution was lower (Supplementary Fig. S3). Based on the density shapes, they could be tentatively fitted with a pair of CoA molecules located symmetrically in a positively charged pocket, except the missing cysteamine group (Supplementary Fig. S8b–d). In addition, we did not observe the densities for the acyl chain of phytanoyl-CoA. Nevertheless, the CoA densities were invisible at the corresponding position with C2 symmetry (Supplementary Fig. S8g). Although the local resolution is relatively lower, we could identify that the adenine ring of ADP is stabilized by Arg383 from TM6 by a hydrogen bond. The 3′-phosphate of ribose forms salt bridges with Lys322 from TM5, Arg379 from TM6 and Lys144′ from TM2′, while Arg379 and Lys144′ also interact with the diphosphate group (Supplementary Fig. S8f). Of note, these key residues are also conserved between ABCD1 and ABCD3 (Supplementary Fig. S6c). To further confirm the binding site of CoA, we generated the alanine substitution variants of these residues and performed ATPase activity assays in the presence of phytanoyl-CoA (Supplementary Fig. S8f). The result indicated that all variants exhibit significantly decreased phytanoyl-CoA-stimulated ATPase activity compared to the WT, indicating that these conserved polar residues are also important for substrate translocation in ABCD3. Compared to the phytanoyl-CoA-bound ABCD3, the binding site of CoA is localized deeper towards the cytosol (Supplementary Fig. S8e). It is possible that the two densities indicated a third CoA-binding site besides the other two sites observed in our structures of phytanoyl-CoA-bound ABCD3 and C22:0-CoA-bound ABCD1. The missing moiety of phytanoyl-CoA might be resulted from the flexibility of the acyl chain, or the hydrolysis by the thioesterase activity of ABCD3. In fact, it was reported that human ABCD1–3^30,31^, as well as the homolog protein CTS in *Arabidopsis thaliana*^32^, possess the thioesterase activity. In this case, BCFA-CoA might be hydrolyzed to CoA and fatty acid at the thioester bond. However, we failed in detecting the reproducible thioesterase activity of ABCD3 after several trials.

Combined with previously reported ABCD1 structures, we proposed a series of snapshots of phytanoyl-CoA fatty acid translocation driven by ABCD3 (Fig. 4c). Firstly, a pair of phytanoyl-CoA molecules approach ABCD3 by the diffusion of CoA moieties into the translocation cavity in a symmetric manner, while the acyl chains stay in the membrane bilayer before entering ABCD3 (State 1). Notably, the Hill coefficient of 1.7 indicated that the two substrate molecules might act cooperatively, binding to the two ABCD3 subunits. However, we do not know exactly whether the two substrate molecules bind simultaneously or sequentially to the pockets under physiological conditions, although we have captured a state where two molecucles of substrates are simultaneously bound. During the entry process, the CoA moiety points towards the peroxisomal space as shown in our substrate-bound structure (State 2). After bound to the next binding sites as indicated by the substrate-bound ABCD1 structure, phytanoyl-CoA fully entered, with its CoA moiety pointing to the cytosol side and the acyl chain embraced by more TM helices, probably crosslinking the two TMDs (State 3). The fully bound substrates trigger conformational change of ABCD3, facilitating the binding of ATP and a further relocation of the CoA moiety deeper towards the cytosol (State 4), as shown in our ATP-bound structure. After the hydrolysis of ATP, phytanoyl-CoA molecules are fully transported. Alternately, it is possible that phytanoyl-CoA is digested into CoA and phytanic acid, after which the phytanic acid is translocated into the peroxisome while CoA is released, possibly, into the cytosol (State 5). In this translocation cycle, the CoA is more specifically recognized and bound in multiple positions in the translocation cavity of ABCD3, representing different intermediate states. The fatty acid chain is pulled by the CoA, and may play a key role in triggering the substrate-induced conformational changes to promote ATP binding and/or hydrolysis.

The present structures of ABCD3 and previous structures of ABCD1 suggested that polar interactions are largely from the CoA moiety, which is shared by ABCD1 and ABCD3 (Fig. 3b). We thus speculate that the translocation pathway driven by the CoA moiety might be similar between ABCD1 and ABCD3. However, substrates of ABCD1 and ABCD3 possess distinct fatty acid chains besides the shared CoA moiety, which are embraced by hydrophobic residues. We thus hypothesize that the different substrate preference of ABCD1–3 should be determined by the hydrophobic residues, which comprise specific binding pockets for different fatty acid chains. However, more structural information on substrate-bound structures of ABCD1–3 is needed to elucidate this hypothesis.

In conclusion, these findings provided not only two states of the BCFA-CoA transport cycle driven by ABCD3, but also biochemical evidence for the binding pattern of acyl acids, revealing a possible translocation cycle with multiple intermediate states applicable for ABCD3 and homologs. These results may shed light on the related human diseases.

## Materials and Methods

### Protein expression and purification

The codon-optimized full-length human *ABCD3* gene and *C. elegans pmp2* were synthesized by GENEWIZ Company. The gene encoding chABCD3 consisting of residues 1–50 of PMP2 and residues 51–659 of human ABCD3 was generated by overlap PCR. Genes of WT protein and mutants were cloned into a pCAG vector with an N-terminal FLAG tag (DYKDDDDK).

For protein expression, the HEK293F cells were cultured in SMM 293T-II medium (Sino Biological Inc.) at 37 °C with 5% CO_2_. When cell density reached ~2.5 × 10^6^ cells per mL, ~1.8 mg plasmids and 4 mg linear polyethylenimines (Polysciences, Inc.) were pre-incubated in 45 mL fresh medium for 15 min, and then the mixture and another 50 mL fresh medium were added into 900 mL cells, followed by 15-min static incubation. The transfected cells were grown at 37 °C for 12–24 h, then 10 mM sodium butyrate (Aladdin) was added, and cultured at 30 °C for 48 h before harvest. Cell pellets were resuspended in the lysis buffer containing 25 mM Tris-HCl (pH 7.5), 150 mM NaCl, 15% glycerol (v/v) after centrifugation at 1500× *g* for 10 min. The suspension was frozen in liquid nitrogen and stored at –80 °C for further use.

The membrane proteins were extracted with 1% (w/v) LMNG (Anatrace), 0.1% (w/v) CHS (Sigma) and 10 mM ATP (Sigma) at 8 °C for 2 h. After ultracentrifugation at 45,000 rpm for 45 min (Beckman Type 70 Ti), the supernatant was incubated with the anti-FLAG M2 affinity gel (Sigma) on ice for 1 h. Then the resin was loaded onto the column and washed three times, each with 10 mL of wash buffer containing 25 mM Tris-HCl, pH 7.5, 150 mM NaCl, 5% glycerol (v/v), 0.02% GDN (w/v) or 0.06% digitonin. Protein was eluted with 6 mL of elution buffer plus 200 µg/mL FLAG peptide. The eluent was concentrated by a 100-kDa cut-off Centricon (Millipore) and then applied to size-exclusion chromatography (Superdex 200 Increase 10/300, GE Healthcare) in wash buffer plus 2 mM DTT. Peak fractions were pooled and frozen in liquid nitrogen or concentrated for EM analysis.

The proteins used for ATPase activity assay were also expressed and purified in the same way except that the detergent in wash buffer and elution buffer was substituted by 0.01% LMNG and 0.001% CHS.

### ATPase activity assay

ATPase activities of WT chABCD3 and mutants were measured by quantitating inorganic phosphate using a modified malachite green-ammonium molybdate method based on a previously described procedure^33^.

All potential substrates of ABCD1 including behenoyl-Coenzyme A (C22:0-CoA, ammonium salt), lignoceroyl-CoenzymeA (C24:0-CoA, ammonium salt), hexacosanoyl-CoenzymeA (C26:0-CoA, ammonium salt), and phytanoyl-Coenzyme A (ammonium salt) were purchased from Sigma-Aldrich and dissolved in 5% (w/v) methyl-β-cyclodextrin (Sigma Aldrich).

To measure the ATPase activities of chABCD3 toward different substrates, a final concentration of 0.05 μM protein was added to the reaction buffer containing 20 mM Tris-HCl, pH 7.5, 50 mM KCl, 1 mM DTT, 0.01% (w/v) LMNG/0.001% (w/v) CHS, and 2 mM MgCl_2_ to 100 μL as one reaction sample. Then, potential substrates were diluted into different concentrations and added into the reaction mixture. The mixture was incubated statically on ice for 10 min and supplemented with 2 mM ATP before reactions were performed at 37 °C for 40 min. Then the amount of released Pi was quantitatively measured and statistical analysis was performed using Origin 2021b (Academic).

### Cryo-EM data collection

To prepare the sample of ATP-bound chABCD3 complex for data collection, the chABCD3-E596Q sample at a concentration of ~7.4 mg/mL was incubated with 10 mM ATP/Mg^2+^ and 1 mM phytanoyl-Coenzyme A for 1 h. An aliquot of 3.5 μL of the sample was applied to glow-discharged Quantifoil R1.2/1.3 300-mesh Cu Holey Carbon Grids. The grids were blotted for 3 s with the blot force 0 and 10-s waiting time, and then plunged into liquid ethane using a Vitrobot Mark IV (FEI) at 8 °C and 100% humidity. Two datasets with a total of 1689 micrograph stacks were automatically collected using EPU software^34^ on a Titan Krios microscope at 300 kV. The microscope was equipped with a K3 Summit direct electron detector (Gatan) and a GIF Quantum energy filter (Gatan), and operated at a nominal magnification of 81,000× with defocus values ranging from −2.0 μm to −1.5 μm. Each stack was exposed in super-resolution mode, resulting in 32 frames per stack, and the total dose for each stack was 54 e^−^/Å^2^. For these stacks, motion correction and dose weighting were performed with patch motion correction with a Fourier cropping factor of 0.5, resulting in a pixel size of 1.07. Meanwhile, the defocus values were estimated using Patch CTF estimation^35^.

To prepare the sample of phytanoyl-CoA-bound ABCD3 complex, the purified chABCD3 concentrated to ~10 mg/mL was mixed with 0.5 mM phytanoyl-CoA and incubated for 1 h on ice. Cryo-EM sample preparation and data collection were conducted using the same methods as those employed for ATP-bound chABCD3. A total of 5028 micrograph stacks were collected.

### Cryo-EM data processing

For the ATP-bound chABCD3 complex datasets, the procedures were performed entirely using cryo-SPARC3.1^35^. For the two datasets, 292,930 and 635,963 particles were automatically picked from dataset 1 and dataset 2. After 2D classification, ab-initial reconstruction and heterogeneous refinement, 74,073 and 81,148 particles were selected, respectively. Then, particles from two datasets were merged. After heterogeneous refinement, 108,252 particles were applied to Non-uniform Refinement with C1 symmetry or C2 symmetry, yielding reconstruction maps at resolutions of 3.3 Å and 3.2 Å, respectively.

For the phytanoyl-CoA-bound chABCD3 dataset, 3892,160 particles were automatically picked. After 2D classification, ab-initial reconstruction and heterogeneous refinement, 96,695 particles were selected. Then selected paticles were applied to Non-uniform Refinement with C2 symmetry, yielding a reconstruction map at a resolution of 2.9 Å.

### Model building and refinement

For the ATP-bound chABCD3 complex, an initial model of chABCD3 was generated by the SWISS-MODEL server^36^, using the cryo-EM structure of human ABCD4 (PDB code: 6JBJ) as the reference. The model was docked into the map of chABCD3 using Chimera. The model of chABCD3 was manually rebuilt in COOT^37^ and refined using Real-space refinement in Phenix^38^ with secondary structure and geometry restraints. The final model contains residues 56–221, 231–338, 347–402, 431–649 in Chain A and Chain B. For NBDs, two prominent densities between two NBDs allowed for the fitting of two ATP molecules. The C1 symmetry map has two extra densities between TM5 and TM6, which can be observed in each TMD, and we fitted a Coenzyme A molecule in each density.

For the phytanoyl-CoA-bound chABCD3 complex, an initial model of chABCD3 was generated by the SWISS-MODEL server, using the EM structure of human ABCD1 (PDB code: 7VZB) as the reference. The model was docked into the map of chABCD3 using Chimera. The model of chABCD3 was manually rebuilt in COOT and refined using Real-space refinement in Phenix with secondary structure and geometry restraints. The final model contains residues 60–407, 428–647 in Chain A and Chain B. Two extra densities between TM5 and TM6 can be observed in each TMD; each density was fitted by a phytanoyl-CoA molecule.

All structures were validated by PHENIX. The UCSF ChimeraX 1.2.5^39^ and PyMOL 2.5.2 (https://pymol.org) were used for preparing the structural figures.

### Supplementary information

Supplementary Information

## Data Availability

The cryo-EM density maps of two structures have been deposited at the Electron Microscopy Data Bank under accession codes: EMD-39871 for phytanoyl-CoA-bound ABCD3, EMD-39703 for ATP-bound ABCD3, and the coordinates have been deposited at the Protein Data Bank under accession codes: 8Z9X for phytanoyl-CoA-bound ABCD3 and 8Z0F for ATP-bound ABCD3.
