# Supplementary material for: Structural insights into human ABCD3-mediated peroxisomal acyl-CoA translocation
Source: Cell Discov. 2024 Sep 3;10:92. doi: 10.1038/s41421-024-00722-8 (PMC11369193; doi:10.1038/s41421-024-00722-8)
Supplement: Supplementary file 1 — Supplementary Information [file 41421_2024_722_MOESM1_ESM.pdf]

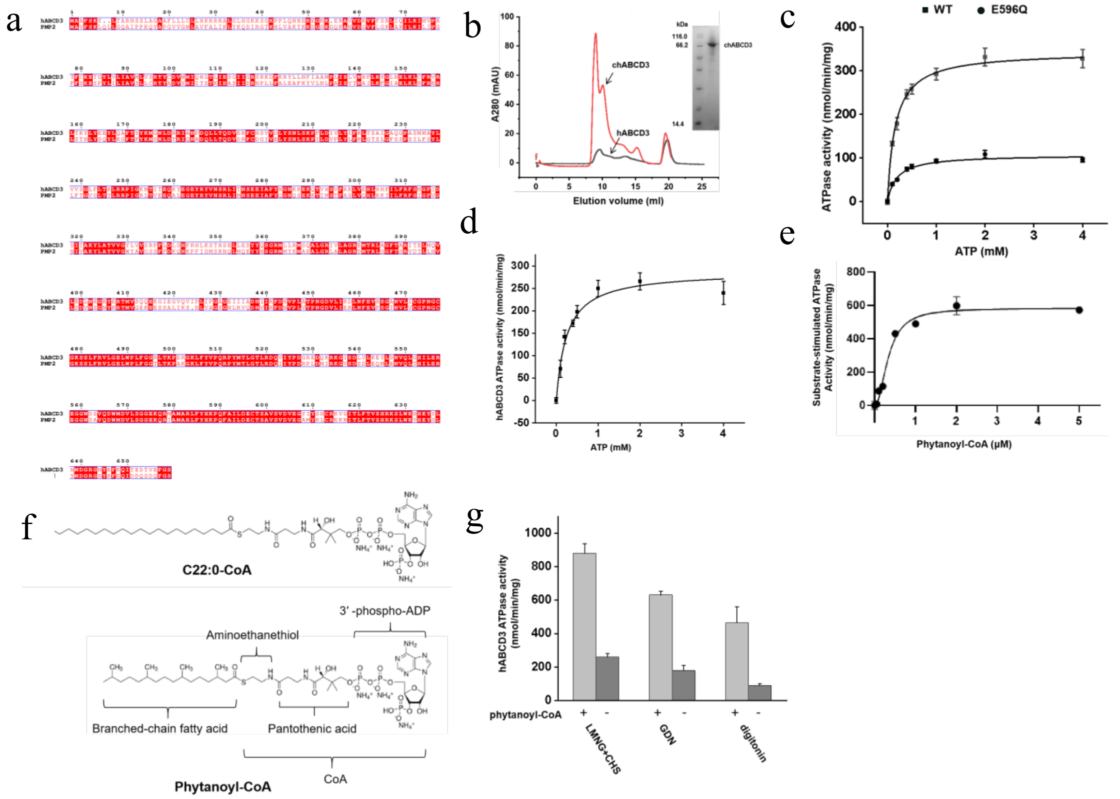

Supplementary Fig. S1 Construction and purification of ABCD3. a Multiple-sequence alignment of human ABCD3 and *Caenorhabditis elegans* PMP2. A chimeric version of ABCD3 was constructed by replacing the N-terminal 50 residues of human ABCD3 with the N-terminal 50 residues from PMP2. **b** Size exclusion chromatography of hABCD3 and chABCD3. The peak fractions of 10 mL for chABCD3 and 10.2 mL for hABCD3. The purified protein was visualized by Coomassie-blue stained SDS-PAGE. **c** ATPase activity of the chABCD3 wild-type (WT) and E596Q mutant purified in LMNG + CHS. The chABCD3 wild-type protein showed a K_m and V_{max} values of 0.16 mM and 343.9 nmol/min/mg protein. The E596Q mutant exhibited an attenuated ATP hydrolysis with K_m and V_{max} values of 0.19 mM and 107.3 nmol/min/mg protein. The data points were fitted with a Michaelis-Menten equation. **d** ATPase activity of the hABCD3 wild-type purified in LMNG + CHS. The wild-type hABCD3 showed K_m and V_{max} values of 0.24 mM and 288.1 nmol/min/mg protein, respectively. The data points were fitted with a Michaelis-Menten equation. **e** Substrate concentration-dependent ATPase activity of chABCD3 in detergent of LMNG + CHS and 2 mM ATP upon addition of phytanoyl-CoA. The data points were fitted with a Hill equation. All data points analyzed above represent means of three independent measurements. Error bars represent the means ± SD. **f** Structure formulas of C22:0-CoA (ammonium salt) and phytanoyl-CoA (ammonium salt). **g** Substrate-stimulated ATPase activity of the chABCD3 wild-type purified

in LMNG + CHS, GDN and digitonin, respectively. 2 mM ATP were added in the presence (+) or absence (-) of 1 μM phytanoyl-CoA, respectively. All data points analyzed above represent means of three independent measurements. Error bars represent the means ± SD.

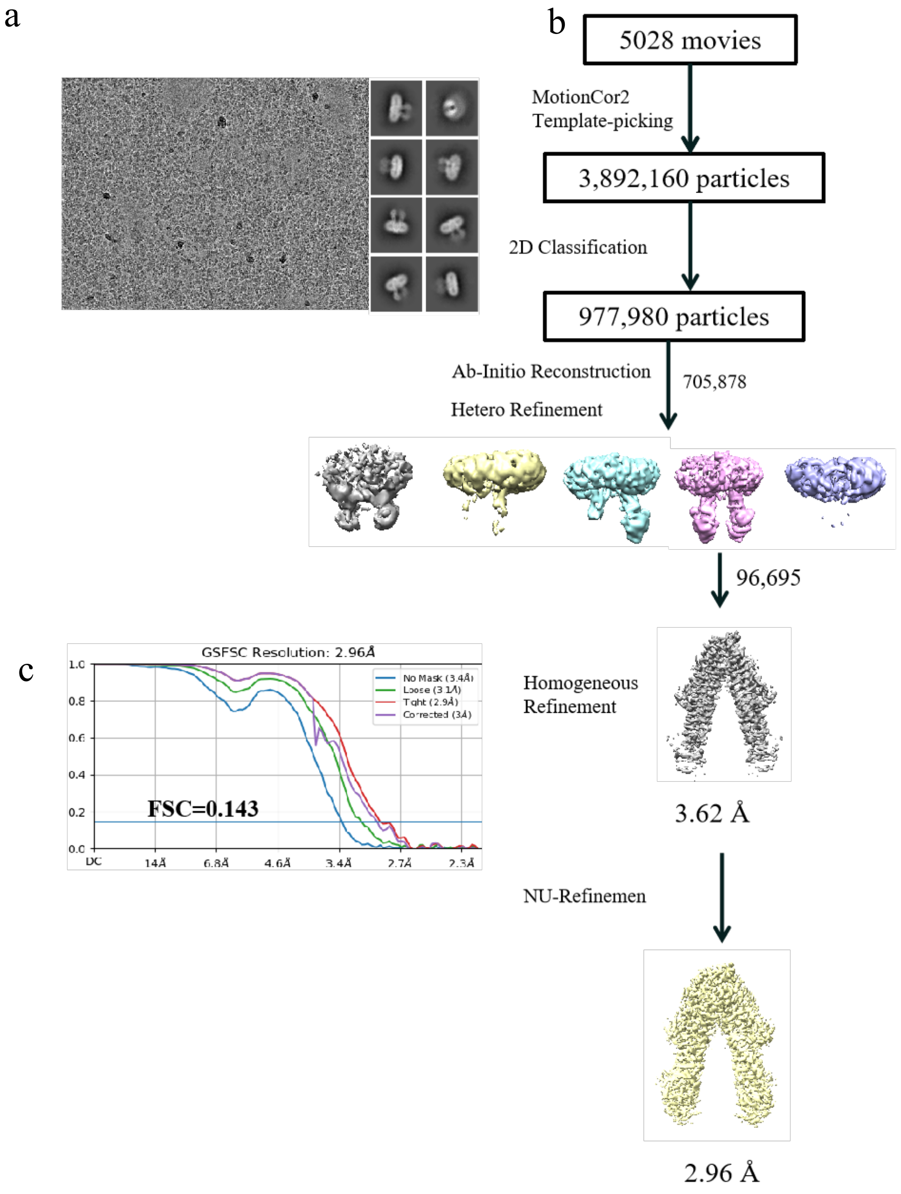

Supplementary Fig. S2 Cryo-EM analysis of phytanoyl-CoA-bound ABCD3. a Representative cryo-EM micrographs and 2D averages. **b** Flowchart for cryo-EM data processing. **c** Gold-standard FSC curve for phytanoyl-CoA-bound ABCD3 map generated using cryoSPARC 3.2.

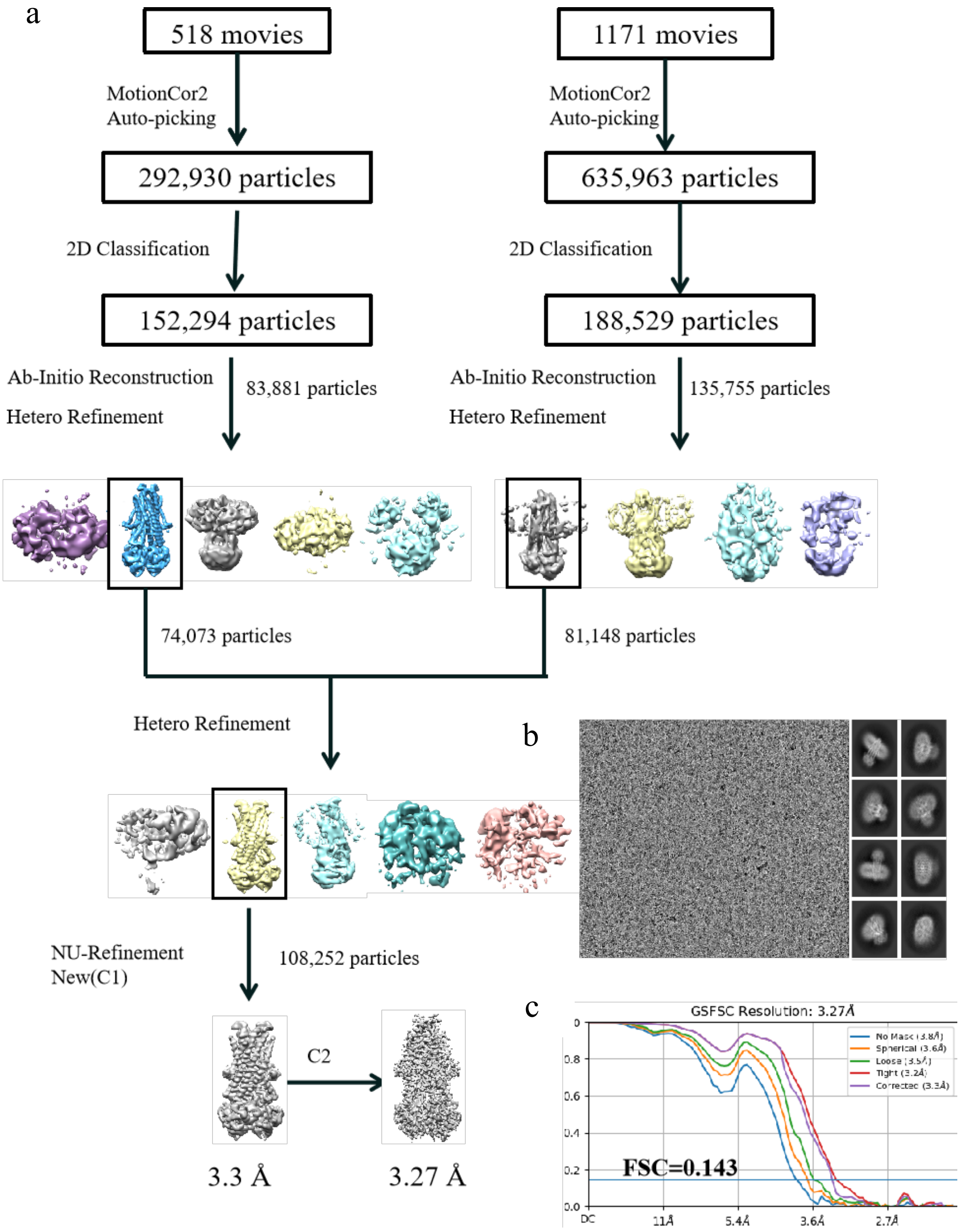

Supplementary Fig. S3 Cryo-EM analysis of ATP-bound ABCD3. a Flowchart for cryo-EM data processing. **b** Representative cryo-EM micrographs and 2D averages. **c** Gold-standard FSC curve for ATP-bound ABCD3 map generated using cryoSPARC 3.2.

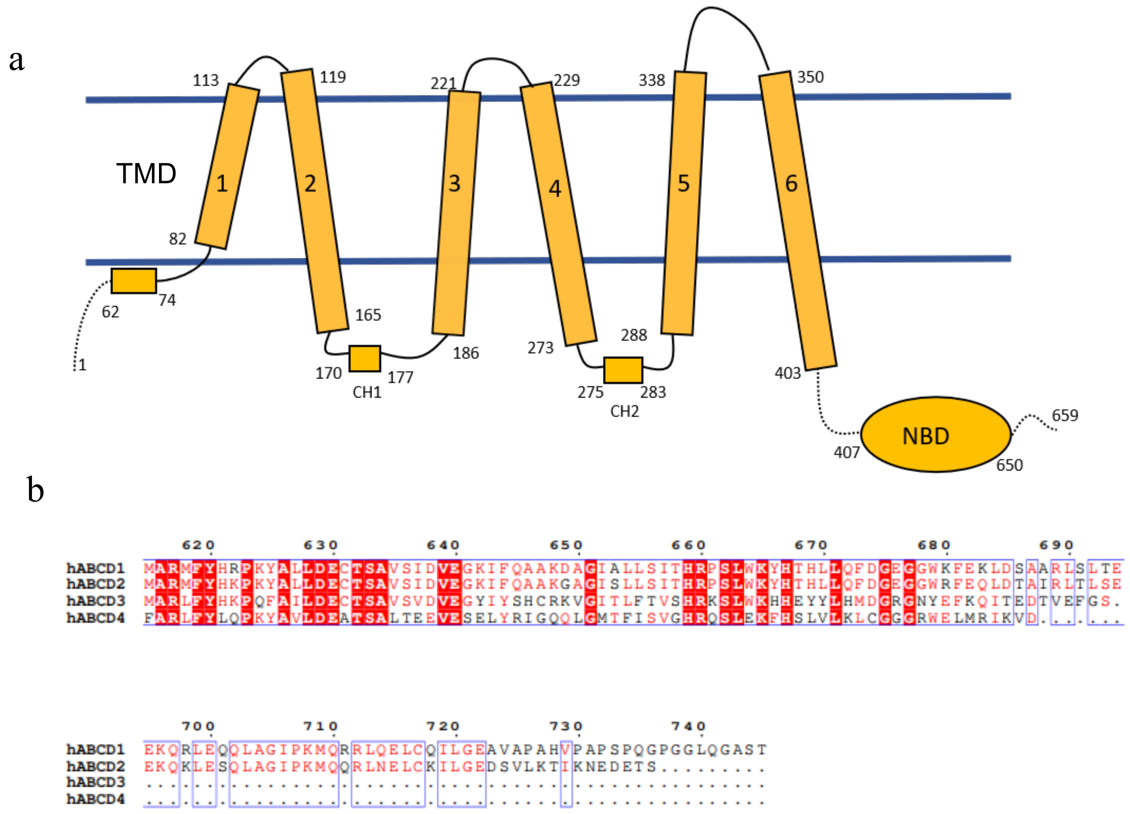

Supplementary Fig. S4 a Topological diagram of ABCD3. The dotted lines represent missing residues in phytanoyl-CoA-bound ABCD3 structure. TMD: transmembrane domain; NBD, nucleotide binding domain; CH, coupling helix. **b** Multiple-sequence alignment of human ABCD subfamily.

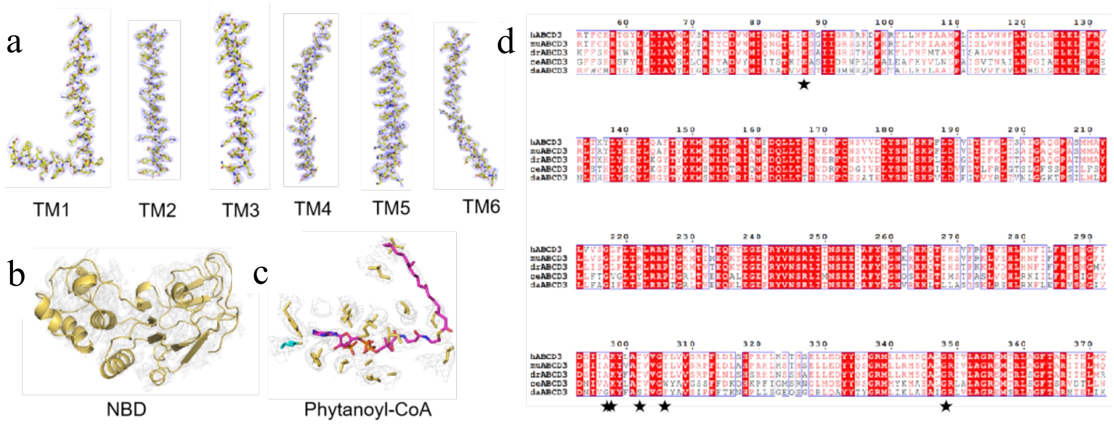

Supplementary Fig. S5 a-c EM densities of representative segments of the structure of phytanoyl-CoA-bound ABCD3. The structure was reconstructed with C2 symmetry, and only one monomer is presented. The density maps of **a** TMs, **b** NBD and **c** phytanoyl-CoA are shown as gray mesh, and are contoured at 8σ, 4σ and 6σ, respectively. **d** Multiple-sequence alignment of human ABCD3 and homologs. Substrate-binding residues of phytanoyl-CoA-bound ABCD3 structure are indicated by an asterisk. h: *Homo sapiens*, mu: *Mus musculus*, dr: *Danio rerio*, ce: *Caenorhabditis elegan*, da: *Drosophila albomicans*.

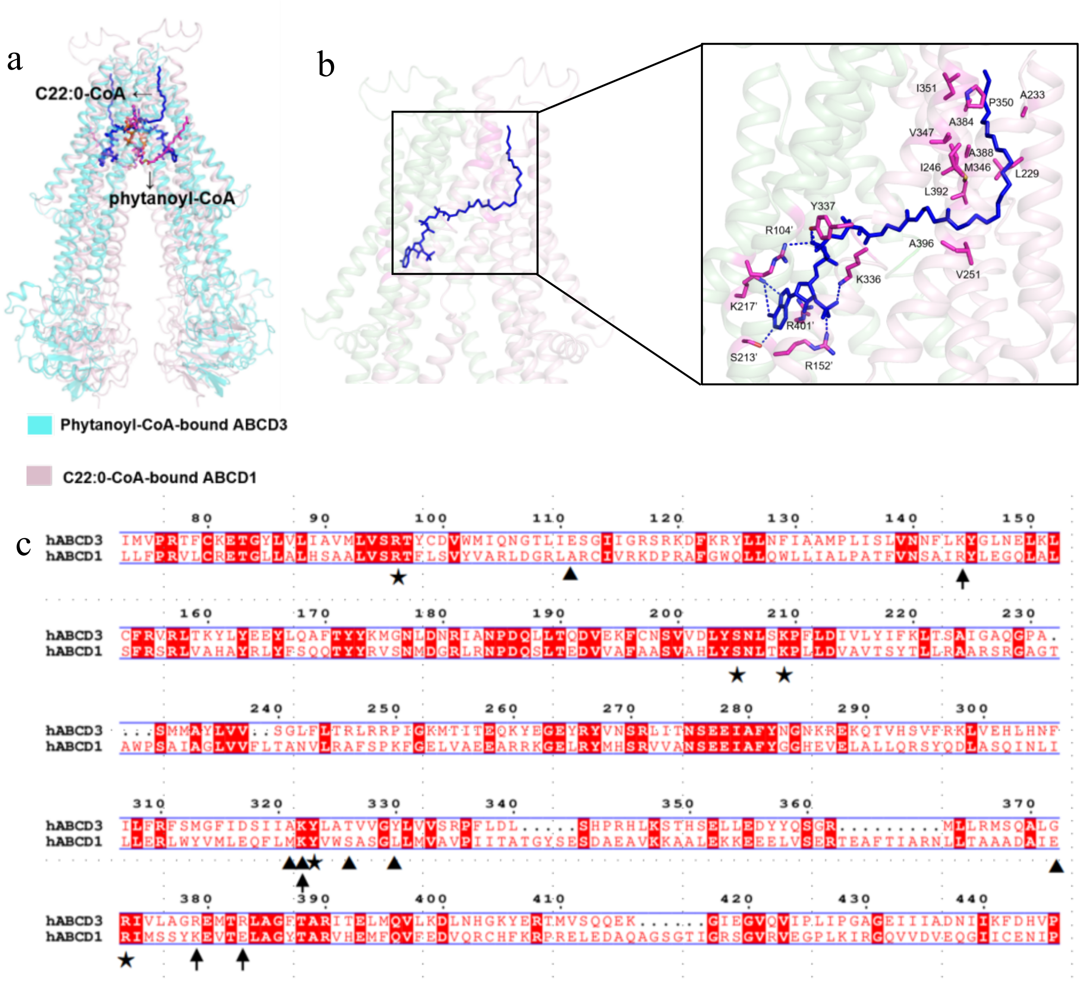

Supplementary Fig. S6 a Superposition of the phytanoyl-CoA-bound ABCD3 against the C22:0-CoA-bound ABCD1. **b** The C22:0-CoA binding residues in ABCD1 are shown as sticks, and hydrogen bonds (≤3.5 Å) and salt bridges (≤4.0 Å) are indicated as blue dotted lines. The hydrophobic residues surrounding the fatty acyl chain within 4.5 Å are shown as sticks. **c** Sequence alignment of human ABCD1 and ABCD3. Substrate-binding residues for CoA portion in the C22:0-CoA-bound ABCD1 structure, the phytanoyl-CoA-bound and ATP/CoA-bound ABCD3 structures are indicated by asterisks, triangles and arrows, respectively.

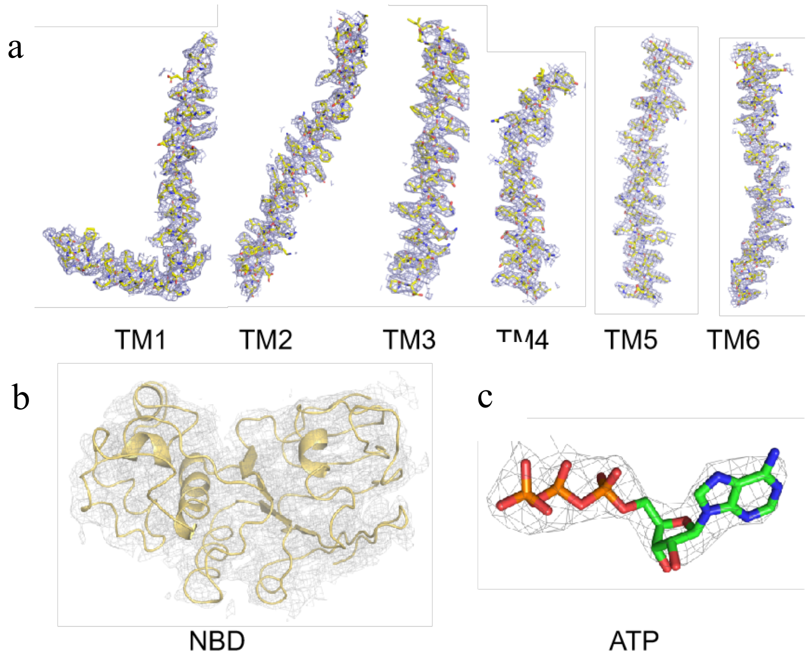

Supplementary Fig. S7 a-c EM densities of representative segments of the structure of ATP-bound ABCD3. The structure was reconstructed with C2 symmetry, and only one monomer is presented. The density maps of **a** TMs, **b** NBD and **c** ATP are shown as gray mesh, and are contoured at 8σ, 4σ and 8σ, respectively.

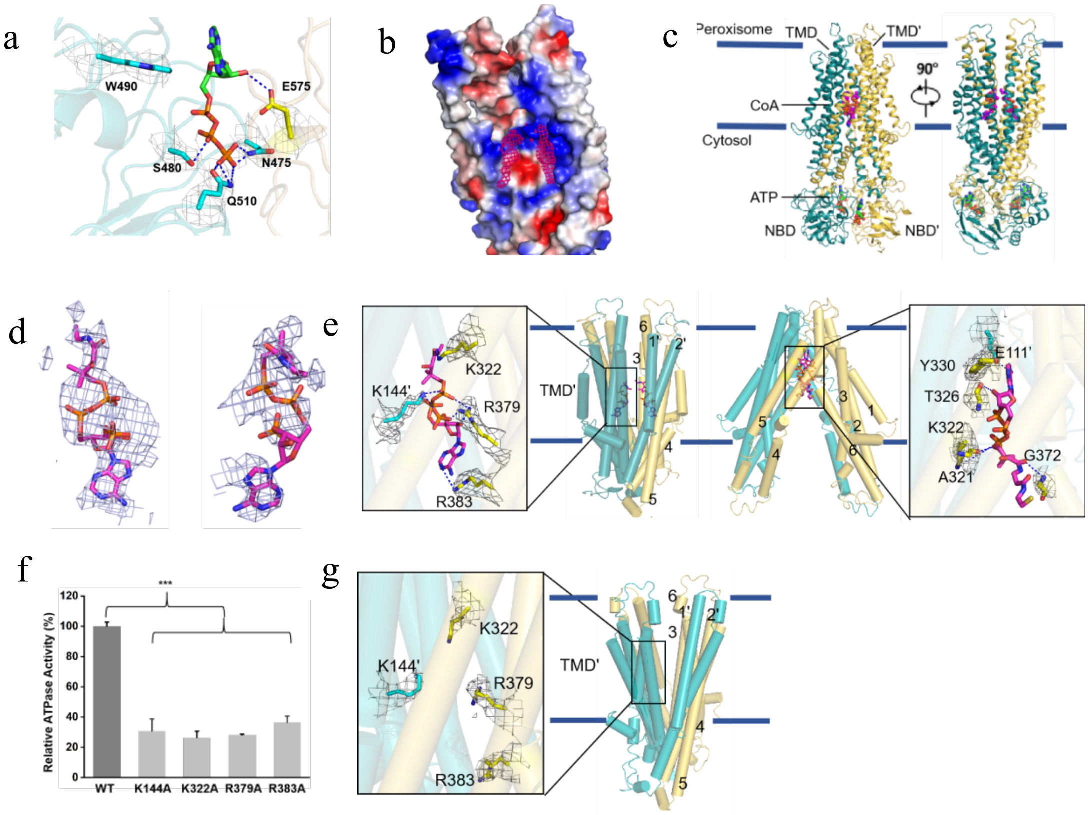

Supplementary Fig. S8 Structure of ATP-CoA-bound ABCD3. **a** ATP molecules are displayed as green sticks. The density maps of coordinating residues shown as gray mesh, and are contoured at 6σ. **b** The electrostatic surface of ATP-bound ABCD3, the two densities are shown. **c** Cartoon representation of ATP-CoA-bound ABCD3. **d** The majority of CoAs fitting into the density maps. The density maps of CoAs are shown as gray mesh, and are contoured at 5σ (left) and 4.5σ (right) corresponding to those in **b**. **e** The CoA binding residues in the ATP/CoA-bound and phytanoyl-CoA-bound structures are shown as sticks, and hydrogen bonds (≤3.5 Å) and salt bridges (≤4.0 Å) are shown as blue dotted lines. The density maps of binding residues shown as gray mesh, are contoured at 5σ. **f** Relative ATPase activities of chABCD3 and mutants in detergent of LMNG + CHS and 2 mM ATP upon addition of phytanoyl-CoA. The relative activity represents the substrate-stimulated activity of chABCD3 or its mutant that harboring a single mutation of residues at the substrate-binding site. Each data point is the average of three independent experiments (n = 3), and error bars represent the means ± SD. T-test analysis of variance is used for the comparison of statistical significance of mutants and wild-type phytanoyl-CoA stimulated ATPase activities. The p value of K144A, K322A, R379A, R383A is 0.00000416, 0.00000229, 0.00000281, 0.00000138, respectively. The p values of <0.05, 0.01, and 0.001 are indicated with *, ** and ***, respectively. **g** The CoA binding

residues in the ATP/CoA-bound structures of C2 map are shown as sticks. The density maps of binding residues shown as gray mesh, are contoured at 5σ.

Supplementary Table S1 Summary of Phytanoyl-CoA-bound ABCD3 cryo-EM data

	Phytanoyl-CoA-bound ABCD3 (EMDB-39871, PDB: 8Z9X)
**Data collection and processing**	
Magnification	81000
Voltage (kV)	300
Camera	Gatan K3 summit
Electron exposure ($e^{-}/Å^2$)	54
Defocus range (μm)	-2.0 to -1.5
Pixel size (Å)	1.07
Symmetry imposed	C2
Initial particle images (no.)	3,892,160
Final particle images (no.)	96,695
Map resolution (Å)	2.96
FSC threshold	0.143
Map resolution range (Å)	2.4-3.81
**Refinement**	
Initial model used (PDB code)	*Ab initio* model
Model resolution (Å)	2.96
FSC threshold	0.143
Model composition	
Non-hydrogen atoms	4673
Protein residues	1318
Ligands	2
**Validation**	
MolProbity score	2.58
Clashscore	10.76
Poor rotamers (%)	0.38
Ramachandran plot	
Favored (%)	92.27
Allowed (%)	7.73
Disallowed (%)	0
Outliers (%)	0
Rotamer outliers (%)	5.4
CaBLAM outliers (%)	4.29

Supplementary Table S2 Summary of ATP-bound ABCD3 cryo-EM data

	ATP-bound ABCD3 (EMDB-39703, PDB: 8Z0F)
**Data collection and processing**	
Magnification	81000
Voltage (kV)	300
Camera	Gatan K3 summit
Electron exposure (e^{-}/Å^2)	54
Defocus range (μm)	-2.0 to -1.5
Pixel size (Å)	1.07
Symmetry imposed	C2
Initial particle images (no.)	928,893
Final particle images (no.)	108,252
Map resolution (Å)	3.27
FSC threshold	0.143
Map resolution range (Å)	2.6-4.3
**Refinement**	
Initial model used (PDB code)	*Ab initio* model
Model resolution (Å)	3.27
FSC threshold	0.143
Model composition	
Non-hydrogen atoms	4471
Protein residues	1318
Ligands	2
**Validation**	
MolProbity score	2.20
Clashscore	12.95
Poor rotamers (%)	0.38
Ramachandran plot	
Favored (%)	89.09
Allowed (%)	10.91
Disallowed (%)	0
Outliers (%)	0
Rotamer outliers (%)	0.51
CaBLAM outliers (%)	6.85
